# Discs no more: the morphology of low-mass simulated galaxies in FIREbox

**DOI:** 10.1093/mnras/staf1847

**Published:** 2025-10-24

**Authors:** José A Benavides, Laura V Sales, Andrew Wetzel, Jorge Moreno, Robert Feldmann, Francisco J Mercado, James S Bullock, Philip F Hopkins, Claude-André Faucher-Giguère, Jonathan Stern, Coral Wheeler, Dušan Kereš

**Affiliations:** Department of Physics and Astronomy, University of California, Riverside, CA 92507, USA; Department of Physics and Astronomy, University of California, Riverside, CA 92507, USA; Department of Physics and Astronomy, University of California, Davis, CA 95616, USA; Department of Physics and Astronomy, Pomona College, Claremont, CA 91711, USA; Carnegie Observatories, Pasadena, CA 91101, USA; Department of Astrophysics, Universität Zürich, Zurich CH-8057, Switzerland; TAPIR, California Institute of Technology, Pasadena, CA 91125, USA; Department of Physics and Astronomy, University of California, Irvine CA 92697, USA; TAPIR, California Institute of Technology, Pasadena, CA 91125, USA; Center for Interdisciplinary Exploration and Research in Astrophysics (CIERA) and Department of Physics and Astronomy, Northwestern University, 1800 Sherman Avenue, Evanston, IL 60201, USA; School of Physics & Astronomy, Tel Aviv University, Tel Aviv 69978, Israel; Department of Physics and Astronomy, California State Polytechnic University, Pomona, Pomona, CA 91768, USA; Department of Astronomy and Astrophysics, University of California, San Diego, La Jolla, CA 92093, USA; Department of Physics, University of California, San Diego, La Jolla, CA 92093, USA

**Keywords:** galaxies: dwarf, galaxies: formation, galaxies: general, galaxies: haloes

## Abstract

We study the morphology of hundreds of simulated central galaxies in the stellar mass range $M_\star =$ 10^7.5^–10^11^  $\rm M_\odot$ from the FIREbox cosmological volume. We demonstrate that FIREbox is able to predict a wide variety of morphologies, spanning from disc-dominated objects to spheroidal galaxies supported by stellar velocity dispersion. However, the simulations predict a strong relation between morphology (degree of rotational support) and stellar mass: galaxies comparable to the Milky Way are often disc-dominated while the presence of stellar discs mostly vanishes for dwarfs with $M_\star < 10^9 ~$$\rm M_\odot$. This defines a ‘morphology transition’ regime for galaxies with $10^9 < M_\star /\rm {M_\odot }< 10^{10}$ in which discs become increasingly common, but below which discs are rare. We show that burstiness in the star formation history and the deepening of the gravitational potential strongly correlate in our simulations with this transition regime, with discs forming in objects with lower levels of burstiness in the last $\sim 6$ Gyr and haloes with mass $\sim 10^{11} ~ \rm {{\rm M}_{\odot }}$ and above. While observations support a transition towards thicker discs in the regime of dwarfs, our results are in partial disagreement with observations of at least some largely rotationally supported gas discs in dwarfs with $M_\star < 10^9$$\rm M_\odot$. This study highlights dwarf morphology as a fundamental benchmark for testing future galaxy formation models.

## 1 INTRODUCTION

Successful formation of discs in cosmological numerical simulations of galaxies required striking a balance between strong stellar feedback and suppressed star formation efficiencies, particularly to prevent overproduction of stars at high redshifts (O. Agertz, R. Teyssier & B. Moore 2011; J. Guedes et al. 2011; F. Marinacci, R. Pakmor & V. Springel 2014). Several studies have highlighted that besides a quiet accretion history, where most stars are born *in situ*, additional fundamental factors to the development of discy structures include avoiding the formation of stars from low angular-momentum gas (M. Steinmetz & J. F. Navarro 1999; C. B. Brook et al. 2011; H. Übler et al. 2014) and coherence in the alignment of the angular momentum of the accreted gas (C. Scannapieco et al. 2009; L. V. Sales et al. 2012; S. Garrison-Kimmel et al. 2018; Z. Hafen et al. 2022).

Almost unanimously, numerical codes have achieved these attributes by generating (or implementing) galactic winds/outflows that help remove some of the gas that would otherwise inevitably cool from the haloes into the galaxies. As numerical treatments to model the physics of the interstellar medium grew more complex and better resolved, predictions suggest that, in low mass haloes, star formation tends to be ‘bursty’, showing relatively large variations in the amount of stars formed in short periods of time (P. F. Hopkins et al. 2014; A. L. Muratov et al. 2015; C.-A. Faucher-Giguère 2018; S. Bose et al. 2019). This feature, while present in most simulations in the dwarfs regime, seems particularly prominent in those sub-grid models that attempt to follow the multiphase nature of the gas (e.g. cooling below a temperature $T \le 10^4$ K), as opposed to adopting an equation-of-state model to describe a hot/cold phase into a single fluid (e.g. V. Springel & L. Hernquist 2003).

Efficient coupling of the feedback energy resulting from such bursty star formation may trigger vigorous outflows and galactic fountains (A. L. Muratov et al. 2015; T. Suarez et al. 2016; D. Martizzi 2020; M. E. Orr et al. 2022; F. Barbani et al. 2023), the formation of bubbles (C. Dalla Vecchia & J. Schaye 2008; B. W. Keller et al. 2014; C.-G. Kim, E. C. Ostriker & R. Raileanu 2017; C. Li et al. 2024) and outflow/inflow cycles – or breathing mode – (K. El-Badry et al. 2016) which overall are disruptive to the coherence of angular momentum in the gas. In the case of cosmological simulations of Milky Way-like (MW-like) galaxies, what ultimately enables the settling and formation of discs is *the halo mass*. As dark matter haloes grow, star formation shifts to less episodic and more continuous along with a deepening of the gravitational potential that ultimately confines the gas from outflows and facilitates the accretion of gas with coherent angular momentum cooling from the hot circumgalactic medium (CGM; e.g. L. V. Sales et al. 2012; B. Clauwens et al. 2018; P. F. Hopkins et al. 2023; J. Stern et al. 2024).

In the regime of dwarf galaxies ($M_{\star } < 10^9 ~ \rm {{\rm M}_{\odot }}$), levels of star formation, temperature of the CGM gas and halo masses may substantially differ from that in MW-like galaxies. This poses an interesting question for modern baryonic treatments that successfully reproduce disc formation in MW-like galaxies: what is the predicted morphology for galaxies of lower mass? The Feedback In Realistic Environments (FIRE) project aims at capturing in detail the physics of galaxy formation in sub-kpc scales (P. F. Hopkins et al. 2018b) which has successfully formed discs in MW-like haloes (S. Garrison-Kimmel et al. 2018) along with a realistic population of satellite galaxies (A. R. Wetzel et al. 2016; S. Garrison-Kimmel et al. 2019). While the model was initially developed for zoom-in cosmological simulations, morphology as a function of mass is a question better suited for full-volume cosmological simulations, which allow for the necessary sampling of environmental effects, halo mass function and diversity of assembly histories. FIREbox, which samples a $\sim$22 Mpc on-a-side cosmological volume using the FIRE baryonic treatment (R. Feldmann et al. 2023) constitutes therefore a valuable tool to tackle galaxy structure in low mass galaxies in simulations that resolve the multiphase nature of the interstellar gas.

Using galaxies from the IllustrisTNG model, S. Tacchella et al. (2019) found that dwarfs formed with a numerical model that correctly predict disc fractions for MW-mass galaxies ends up with less disc-dominated structure than their MW-mass counterparts. Moreover, a comparison with data from the Galaxy And Mass Assembly (GAMA) survey (I. K. Baldry et al. 2010; L. S. Kelvin et al. 2012; R. Lange et al. 2016) suggests that the trend closely follows the morphological distribution of real galaxies in the $M_{\star } \sim 10^{9.5} \rm -10^{11.5} ~ {\rm M}_{\odot }$ range if one assumes that dwarf irregulars in the GAMA survey are dispersion-dominated. Limitations in the resolution of the simulations prevented to further explore the regime of even less massive dwarfs. More recently, B. M. Celiz et al. (2025) identified a clear stellar mass–morphology relation where low mass dwarfs are increasingly more dispersion-dominated using the higher-resolution TNG50 run (see also G. Zeng et al. 2024).

In this paper, we use the FIREbox simulation to study the morphology of galaxies predicted as a function of mass for a volume-complete sample simulated with a detailed interstellar medium (ISM) model that follows the multiphase nature of the gas. Previous studies have hinted at the possibility that the FIRE-2 model results in dwarf galaxies that are kinematically more dispersion-dominated than observed, for example, regarding the kinematics of H i gas (K. El-Badry et al. 2018b). However, these conclusions have primarily been based on a limited set of zoom-in simulations, rather than on a large-volume cosmological run. More recently, C. Klein et al. (2025) suggests that the shapes of dwarf galaxies in FIREbox are rounder than in observations, highlighting a potential tension between the observed morphology in this regime and predictions from this simulation. In this paper, we investigate the role of angular momentum support and explore the physical mechanisms that give raise to a morphological transition with stellar mass. In particular, we concentrate on the halo and stellar mass scale where rotationally supported structures transition from rare to most common, which for the FIREbox simulation occur at $M_{\star } \sim 10^{9} \rm -10^{10} ~ {\rm M}_{\odot }$. Our paper is organized as follows. In Section 2, we briefly describe the simulation and some details of our sample of simulated galaxies. In Section 3, we present our main results in the morphological transition and formation of the disc structure in different stellar mass ranges. In Section 4, we present the scenario of disc formation through a wet coplanar merger, as a possible path of discs formation in low-mass galaxies. Finally, we present a general discussion and conclusions in Section 5.

## 2 SIMULATIONS AND METHOD

The simulations in this work were run using the FIRE-2 model (P. F. Hopkins et al. 2018b) coupled to the meshless finite-mass gizmo code (P. F. Hopkins 2015). Gravity is calculated with a modified version of the parallelization and tree gravity solver of gadget-3 (V. Springel 2005). The FIRE-2 model incorporates gas cooling down to $\rm {10 ~ K}$, which naturally leads to the formation of a multiphase interstellar medium (ISM). Once a gas cell becomes eligible for star formation, it is eligible to convert a star particle on a local free-fall time. Stellar feedback associated to radiation, winds, and supernova explosions is modelled by thermal or momentum input depending on whether the relevant scales are resolved (see P. F. Hopkins et al. 2018a, 2020a, for more details). The FIRE-2 model has been validated through numerous studies on galaxy formation and evolution, including satellite galaxy properties, MW-like morphologies, numerical effect testing, and also structural properties of dwarf, all of particular relevance to this study (see e.g. A. R. Wetzel et al. 2016; T. K. Chan et al. 2018; S. Garrison-Kimmel et al. 2018; P. F. Hopkins et al. 2018b).

We use two different kinds of data from the Feedback In Realistic Environments (FIRE^1^) project (P. F. Hopkins et al. 2014, 2018b), (i) FIREbox (R. Feldmann et al. 2023) and (ii) detailed individual zoom-in runs with halo masses $\sim 10^{11}$$\rm M_\odot$ (*m11* runs) all reaching higher resolutions than FIREbox, as detailed in Table 1. The cosmological parameters used in each simulation are slightly different and are described in their respective sections. We briefly describe both data products below.

**Table 1.** Summary of simulations used in this work. First row correspond to our main sample from the FIREbox cosmological box. The subsequent lines describe the sample of high-resolution *m11* zoom-in runs from the FIRE-2 suite. The first column: the simulation name, the second column: details of the physical model (no-MD: no-metal diffusion, MD: with metal diffusion, MD + CR: metal diffusion and cosmic rays, uvb: ultraviolet background), the third column: the initial resolution per gas cell in $\rm {M_\odot }$, the fourth and fifth column are the halo and stellar mass, respectively. The sixth column indicate the main reference of each simulation + their respective ultraviolet background model implemented: (1): R. Feldmann et al. (2023), (2): P. F. Hopkins et al. (2018b), (3): K. El-Badry et al. (2018a), (4): P. F. Hopkins et al. (2020b), *: C.-A. Faucher-Giguère et al. (2009), **: C.-A. Faucher-Giguère (2020).

Name	Details	Res. $m_b/\rm {{\rm M}_{\odot }}$	$M_{\rm vir}/\rm {{\rm M}_{\odot }}$	$M_\star /\rm {{\rm M}_{\odot }}$	Ref.
FIREbox	FIRE-2	63000	$\sim 10^{9}-10^{12.4}$	$10^{7.5}-10^{11}$	(1)*
m11q	MD	880	$1.3 \times 10^{11}$	$3.4 \times 10^{8}$	(2)*
m11h	UVB	880	$1.5 \times 10^{11}$	$1.1 \times 10^{8}$	(3)**
m11q	UVB	880	$1.2 \times 10^{11}$	$2.3 \times 10^{8}$	(2)**
m11d	UVB	880	$2.2 \times 10^{11}$	$7.6 \times 10^{8}$	(3)**
m11e	UVB	880	$1.3 \times 10^{11}$	$4.4 \times 10^{8}$	(3)**
m11b	MD + CR	2100	$3.9 \times 10^{10}$	$4.2 \times 10^{7}$	(4)*
m11a	no-MD	2100	$4.1 \times 10^{10}$	$1.1 \times 10^{8}$	(2)*
m11c	no-MD	2100	$1.4 \times 10^{11}$	$8.2 \times 10^{8}$	(2)*
m11d	no-MD	7100	$3.2 \times 10^{11}$	$4.0 \times 10^{9}$	(4)*
m11i	no-MD	7100	$7.8 \times 10^{10}$	$9.1 \times 10^{8}$	(4)*

### 2.1 FIREbox

FIREbox follows the evolution of a 22.1 cMpc$^3$ volume set-up initially with a total of $2 \times 1024^3$ of gas elements and dark matter particles. As all cosmological-volume simulations, it naturally resolves a myriad of environments and halo formation histories, offering a path to study galaxy properties and evolution with a statistical base. The mass resolution of baryons is $m_{\rm bar} = 6.3 \times 10^4 ~ \rm {M_\odot }$ and $m_{\rm DM} = 3.3 \times 10^5 ~ \rm {M_\odot }$ for dark matter particles with a force resolution softening of 12 pc for stars and 80 pc for dark matter. The force softening of gas cells is adaptive, reaching a minimum of 1.5 pc in the dense ISM. FIREbox follows the evolution of multiphase gas, modelled following the FIRE-2 framework. In this simulation, the initial conditions are generated at redshift $z = 120$ using the MUlti-Scale Initial Conditions (music; O. Hahn & T. Abel 2011) code. It assumes a set of cosmological parameters consistent with the Planck Collaboration XIII (2016) measurements: $\Omega _{\rm m} = \Omega _{\rm dm} + \Omega _{\rm b} = 0.3089, \Omega _{\rm b} = 0.0486$, cosmological constant $\Omega _{\rm \Lambda } = 0.6911$, Hubble constant $\rm { H_0 = 100 \, h \, km \, s^{-1} \, Mpc^{-1} }$, $h = 0.6774$, $\sigma _8 = 0.8159$, and spectral index $n_s = 0.9667$. Previous studies of FIREbox focused on dwarf galaxies show the potential of FIRE-2 model in this new large cosmological volume (J. Moreno et al. 2022; E. Cenci et al. 2024; C. Klein et al. 2025; F. J. Mercado et al. 2025).

### 2.2 Individual low-mass galaxies zoom-in runs

To explore the impact of numerical resolution, we compare FIREbox results with 10 zoom-in runs from the FIRE project, with central host dark matter haloes with masses of $M_{h} \sim 10^{11}~{\rm M}_{\odot }$ (hereafter m11’s, see Table 1). Note that the resolution per (baryonic) particle of all these zoom-in runs is $\sim 8$–70$\times$ better than that achieved in FIREbox. In comparison with FIREbox, the zoom-in runs use a similar set of cosmological parameters, with small variations: $\Omega _{\rm m} = 0.266-0.31$, $\Omega _{\rm \Lambda } = 0.69-0.734$, $\Omega _{\rm b} = 0.044-0.048$, $\sigma _8 = 0.801-0.82$, and $n_s = 0.961$–0.97. However, at the scale of individual galaxies, variations due to differing cosmological parameters are small compared to run-to-run stochasticity. For details, please see the Public Data release (A. Wetzel et al. 2023). Also, small variations of the physics are included between runs, as follows:

(i) Metal diffusion (MD) and no-metal diffusion (no MD), refer to an explicit treatment (or not) for the diffusion of metals (e.g. P. F. Hopkins 2017; P. F. Hopkins et al. 2018b). (ii) Addition of cosmic rays (CRs) feedback (T. K. Chan et al. 2019; P. F. Hopkins et al. 2020b), which can drive multiphase winds, reduce star formation rates (SFRs; mostly in low-mass galaxies) and modify the phase structure of the CGM, and (iii) the higher resolution runs of K. El-Badry et al. (2018a) and P. F. Hopkins et al. (2018b) but implementing the ultraviolet background (UVB) model of C.-A. Faucher-Giguère (2020), that permeates the intergalactic medium, keeping it ionized following the epoch of reionization. The other zoom-in used here implement the previous UVB model of C.-A. Faucher-Giguère et al. (2009). These runs have all been introduced in several previous works (see e.g. T. K. Chan et al. 2018; I. Escala et al. 2018; K. El-Badry et al. 2018a; P. F. Hopkins et al. 2018b, 2020b, 2023; E. D. Jahn et al. 2019).

### 2.3 Sample of simulated galaxies

We select all central galaxies from FIREbox (defined as the most massive centrally located galaxy in a dark matter halo) in the stellar mass range $M_{\star } = [10^{7.5}-10^{11}] ~ \rm {{\rm M}_{\odot }}$. The lower stellar mass bound represents ∼500 stellar particles. From the *m11* zoom-in runs, we select always the main (central) object only. No satellites are included in our samples. In FIREbox, haloes and sub-haloes (galaxies) are identified using the amiga Halo Finder (AHF; S. R. Knollmann & A. Knebe 2009). While, in the FIRE zoom-in sample, galaxies and their haloes are identified using rockstar (P. S. Behroozi, R. H. Wechsler & H.-Y. Wu 2013), with an extended algorithm to append the stellar and gas elements corresponding to each structure found in dark matter. Throughout this paper, virial quantities are measured using the radius within which the average density is 200 times the critical density of the Universe. Stellar masses are computed within the galaxy radius $r_{\rm gal}$, defined here as the radius that encloses 90 per cent of the stellar particles within the dark matter halo, associated to the central galaxy. The centre of each object is defined as the most bound particle. In order to characterize the morphology of the simulated galaxies we use the $\kappa _{\rm rot}$ parameter (L. V. Sales et al. 2012), defined as a ratio that compares the energy in rotational support to the total kinetic energy of the stellar particles in a galaxy. More specifically,


(1) \begin{eqnarray*}
\kappa _{\rm rot} = \frac{K_{\rm rot}}{K}=\frac{1}{K} \sum \,\, \frac{1}{2} m \left(\frac{j_z}{R} \right)^2 \ \ ,
\end{eqnarray*}

where *K* is the total kinetic energy of the galaxy calculated by using the stellar particles within the $r_{\rm gal}$, $j_z$ is the *z*-component of the angular momentum of each stellar particle (having rotated the galaxy, such that the total angular momentum points in the *z* direction), *m* denotes mass, *R* is their cylindrical galactocentric distance and the sum is over stars within the galaxy radius. Typically, high values of $\kappa _{\rm rot} \ge 0.5$ are indicative of rotationally supported systems, such as discs, while lower values of $\kappa _{\rm rot} \le 0.35$ are more characteristic of dispersion-dominated objects, such as traditional bulges. Intermediate values typically correspond to galaxies that contain both bulge and disc components or to dynamically hotter discs that are partially supported by dispersion.

**Figure 1.**
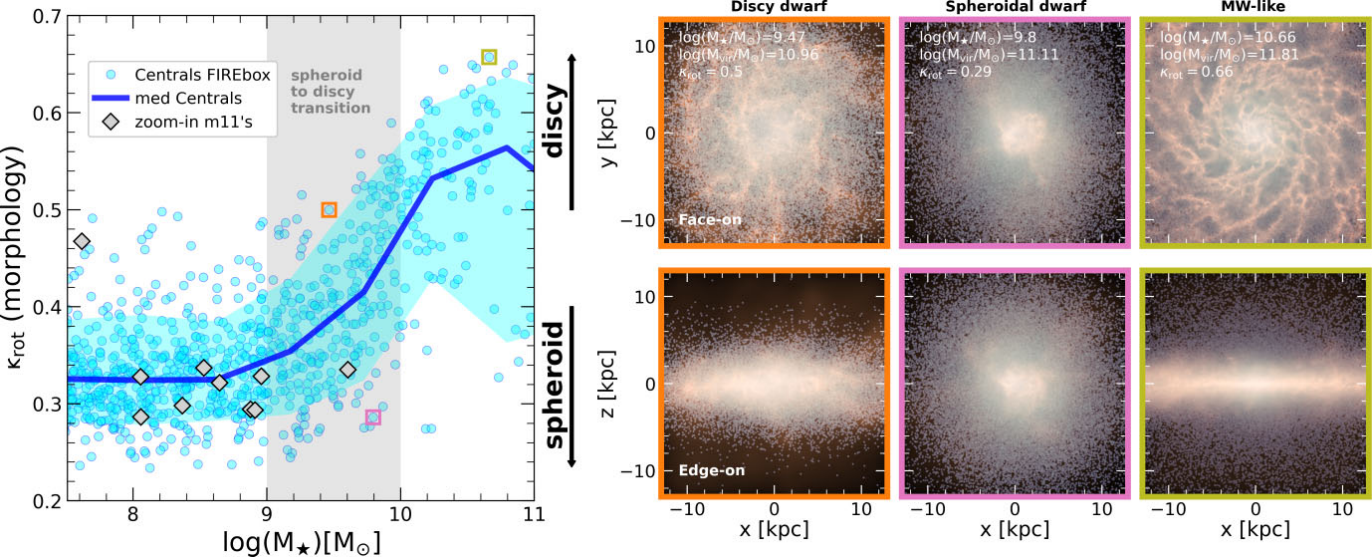
Left: morphology ($\kappa _{\rm rot}$) as a function of stellar mass for all central galaxies in the FIREbox simulation (cyan circles). The median of the distribution is shown by the blue solid line, and the shaded cyan region encloses the 10th–90th percentiles. Morphology depends strongly on stellar mass: dwarfs are spheroidals while MW-like galaxies are mostly disc-dominated. We highlight in grey shading the ‘transition region’ $M_{\star } = [10^9-10^{10}] ~ \rm {{\rm M}_{\odot }}$ where discs begin to form to later dominate galaxy structure. Higher resolution *m11* zoom-in runs are shown in graey diamonds, which agree well with the FIREbox morphologies. Right: examples of galaxies with varied morphology in FIREbox. The top and bottom rows show face-on and edge-on views of stars and gas for a discy dwarf (left), a spheroid-dominated dwarf (middle), and a disc-dominated MW-like galaxy (right). These objects are highlighted in colour open squares on the left panel. The images were generated by using the py-sph Viewer library (A. Benítez-Llambay 2017).

The left panel of Fig. 1 shows the $\kappa _{\rm rot}-M_\star$ relation of our sample of simulated galaxies. The cyan circles represent the sample of central galaxies from FIREbox, and the blue-thick solid line indicates the median $\kappa _{\rm rot}$ at a given stellar mass (shadow region encloses the 10th–90th percentiles). Gray diamonds indicate the morphology of the zoom-in m11’s runs. Encouragingly, FIREbox spans a much wider range of morphology than the selected zoom-in runs, highlighting the relevance of sampling different environments and assembly histories. We showcase three examples on the right panel of Fig. 1, selected to have different masses and morphologies (displayed from left to right): a discy and a spheroidal low-mass galaxies, and a MW-like disc-dominated galaxy. These FIREbox galaxies are highlighted on the left panel using coloured open squares.

The left panel in Fig. 1 highlights a clear trend predicted in the morphology of simulated galaxies: while discs are frequent and dominant for MW-like galaxies ($M_\star \ge 10^{10.5}~ \rm {{\rm M}_{\odot }}$, large $\kappa _{\rm rot}$ values), they become progressively rarer for lower mass galaxies, to the point that no stellar discs ($\kappa _{\rm rot} > 0.5$) are predicted in the simulation for dwarfs with $M_\star < 10^8~ \rm {{\rm M}_{\odot }}$. This leads to a ‘transition’ regime in the stellar mass range $M_\star \sim [10^9-10^{10}]~ \rm {{\rm M}_{\odot }}$ (grey shaded region) where the frequency of rotationally supported systems drops until they fully disappear below $M_\star \sim 10^9~ \rm {{\rm M}_{\odot }}$. Note that the higher resolution zoom-in runs agree with the expected median trend of FIREbox, suggesting that this trend is not driven by numerical resolution in the FIREbox simulation. We now investigate what factors play a role in this morphological transition in objects simulated with a fixed galaxy formation model that successfully reproduces healthy and observationally compatible discs at scales of the MW.

## 3 THE ORIGIN OF THE MORPHOLOGICAL TRANSITION IN THE FIRE SIMULATED LOW-MASS GALAXIES

### 3.1 Halo spin

**Figure 2.**
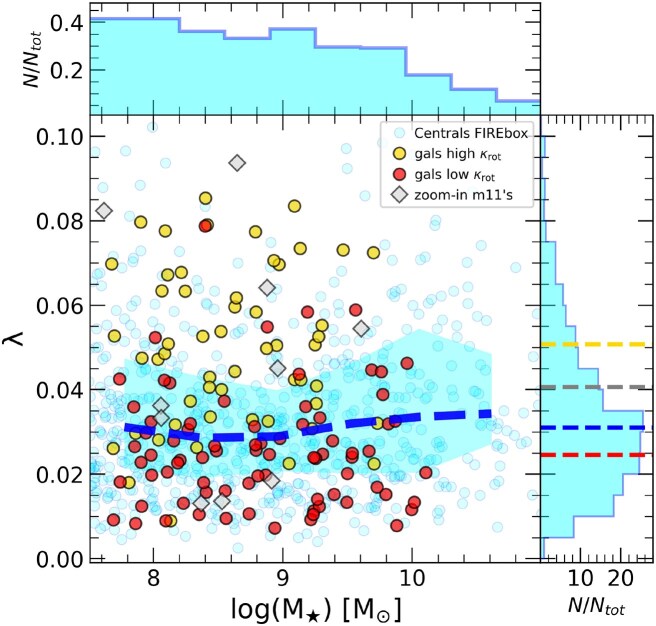
Halo dimensionless spin parameter ($\lambda$) as a function of stellar mass for all central galaxies in FIREbox simulation (cyan circles), along with the median in blue dashed and shaded regions enclose the 25th–75th percentiles. Yellow and red circles indicate the location of low-mass galaxies ($M_{\star } = [10^{7.5}-10^{10}] ~ \rm {{\rm M}_{\odot }}$) whose stars are most (high-$\kappa _{\rm rot}$) and least (low-$\kappa _{\rm rot}$) rotationally supported, selected as the objects above and below the 90th and 10th percentile in the $\kappa _{\rm rot}-M_{\star }$ relation in Fig. 1. The histograms in each axis represent the distribution of stellar mass and spin of the samples, along with their median $\lambda$ (coloured dashed lines). The median (and the 25th–75th percentiles) of halo spin for all central galaxies is $\lambda _{\rm all}=0.031^{+0.014}_{-0.010}$, independent of $M_\star$ as expected. Morphology for low-mass dwarfs shows some dependence on the spin parameter, with high-$\kappa _{\rm rot}$ galaxies having $\lambda _{\rm {high}-\kappa _{\rm rot}}=0.051^{+0.019}_{-0.014}$ while the low-$\kappa _{\rm rot}$ sub-sample shows $\lambda _{\rm {low}-\kappa _{\rm rot}}=0.025^{+0.008}_{-0.009}$. We include the 10 zoom-in *m11*’s FIRE runs with grey diamonds, with $\lambda _{\rm {m11^{\prime }s}}=0.041^{+0.021}_{-0.018}$, which agree with the overall FIREbox distribution.

To quantify the influence of dark matter halo dynamics on the stellar component in Fig. 2 we present the dimensionless halo spin parameter ($\lambda$) as a function of stellar mass for all simulated central galaxies. We have calculated the $\lambda$ halo spin following J. S. Bullock et al. (2001),


(2) \begin{eqnarray*}
\lambda = \frac{J}{\sqrt{2} M_{\rm vir} V_{\rm vir} r_{\rm vir} } \ ,
\end{eqnarray*}

where *J* is the total angular momentum of dark matter particles within the virial radius ($r_{\rm vir}$). The distribution of the halo spin parameter of all central galaxies in FIREbox (cyan circles) presents a median value of $\lambda _{\rm all}=0.031^{+0.014}_{-0.010}$, independent of $M_\star$. The *m11* zoom-in runs (grey diamonds) show a median of $\lambda _{\rm {m11^{\prime }s}}=0.041^{+0.021}_{-0.018}$ which is both consistent with FIREbox and J. S. Bullock et al. (2001). As expected from Lambda cold dark matter ($\Lambda$CDM), we confirm that the halo spin does not vary noticeably over the stellar mass range probed in our sample.

It has been shown that the halo spin parameter plays a negligible role in the morphology of galaxies with masses comparable to the MW using the FIRE-2 model (S. Garrison-Kimmel et al. 2018), a trend reported also in other simulations (e.g. L. V. Sales et al. 2012). However, for low mass galaxies, halo spin can play a more significant role (V. Rodriguez-Gomez et al. 2017). Here, we explore the link between $\lambda$ and morphology for galaxies in FIREbox with $M_\star = 10^{7.5}-10^{10} ~ \rm {{\rm M}_{\odot }}$. Yellow and red circles in Fig. 2 show the spin parameter sub-populations with excess rotational support (high-$\kappa _{\rm rot}$) and a deficit of rotational support (low-$\kappa _{\rm rot}$), selected as objects above of 90th percentile and below 10th percentile, in the $\kappa _{\rm rot}-M_\star$ relation, respectively.

We find a weak (but statistically significant) trend of morphology with halo spin for our low-mass dwarfs. The median $\lambda$ values for high (yellow) and low (red) $\kappa _{\rm rot}$ samples are indicated on the vertical histogram, and suggest that dwarfs with more rotation support in their stellar components tend to occupy haloes with larger spins. This trend seems to suggest that halo spin does play a role in defining the morphology or rotational support of the baryons in dwarf galaxies. However, the halo spin-galaxy kinematics relationship is complex (e.g. F. Jiang et al. 2019; H. Yang et al. 2023), and we cannot rule out a link that is imparted later. In such scenario, the correlation between morphology and halo spin arises due to the same baryonic processes spinning up the gas and stars in galaxies *conjunctively* with the dark matter component. Such analysis would require the availability of a matched sample of the same haloes run in the dark matter-only version, which we defer to future work.

With that caveat in mind, the median spin of high-$\kappa _{\rm rot}$ dwarfs is $\lambda _{\rm {high}-\kappa _{\rm rot}}=0.051^{+0.019}_{-0.014}$ (uncertainties corresponding to the 25th–75th percentiles), while the median for low-$\kappa _{\rm rot}$ dwarfs is $\lambda _{\rm {low}-\kappa _{\rm rot}}=0.025^{+0.008}_{-0.009}$. Despite this median trend (which agrees with findings from a different galaxy formation model presented in V. Rodriguez-Gomez et al. 2017), there is substantial overlap in the spin of dwarfs with most and least rotation support, highlighting that while halo spin can play some role in defining the rotation support in low-mass galaxies, it is not sufficient to predict the final morphology of the simulated dwarfs.

### 3.2 Burstiness and the depth of the gravitational potential

**Figure 3.**
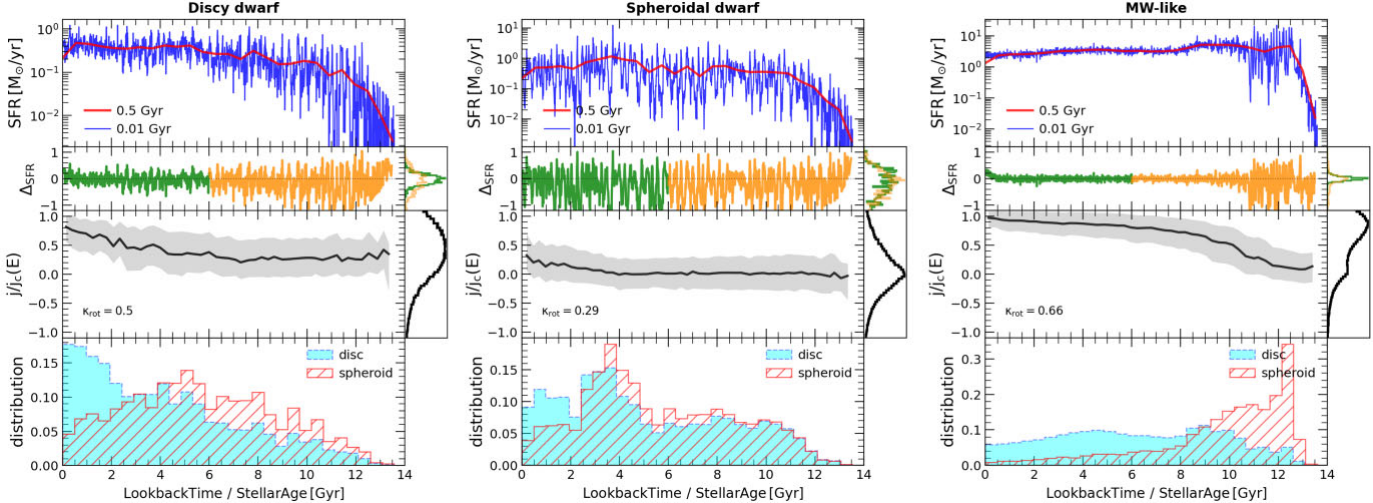
Several evolutionary properties for the three example galaxies in Fig. 1, discy dwarf (left), spheroidal dwarf (centre), and MW-like (right). First row: star-formation rate (SFR) as a function of lookback time averaged in two time-scales, 500 Myr in red, and 10 Myr in blue. Second row: the logarithmic difference between the SFR curves averaged in 10 Myr with respect to the 500 Myr one, $\rm {\Delta _{SFR}}$. The distribution of $\rm {\Delta _{SFR}}$ above and below 6 Gyr are shown in the right box with green and orange colours, respectively. Third row: median of the stellar circularities ($\epsilon = j/j_c (E)$), and their distribution in the vertical histogram. Fourth row: distribution of stellar particles at present-day associated with the disc ($\epsilon > 0.5$ in blue) and spheroid ($\epsilon < 0.5$ in red) components in each galaxy. For the MW-like galaxy, burstiness subsides $\sim 10$ Gyr ago and a stellar disc begins to grow with circularities $j/j_c \sim 1$. For the dwarfs, burstiness continues throughout their evolution, with the spheroid-dominated one showing larger bursts (wider $\rm {\Delta _{SFR}}$ variations).

In the case of MW-like galaxies simulated with the FIRE model, A. L. Muratov et al. (2015) and S. Yu et al. (2021, 2023) show that the SFR evolves with time, transforming from ‘bursty’ at early redshifts – coincidental with the assembly of the spheroidal and thick-disc component – to a more stable, steady SFR at late times that promotes the formation and settling of the thin disc. We show in Fig. 3 the SFR evolution (top panel) for the three FIREbox galaxies presented in the right panel of Fig. 1. The right-most column corresponds to the MW-like analogue and shows a similar behaviour as found in S. Yu et al. (2023) using FIRE-2 zoom-in runs.

Burstiness can be quantified by comparing the fluctuations of the SFR in two different time-scales. Following J. A. Flores Velázquez et al. (2021), we show the SFR averaged over short time-scales (10 Myr, blue curve) along with that averaged over longer time scales (500 Myr, red curve). For the specific case of the MW-mass object (left column), we see that the SFR is characterized by rapid fluctuations before a lookback time $\sim 10$ Gyr, followed by a quiet-down phase onwards where the short-term averaged SFR coincides nicely with the SFR averaged over longer timescales.

We define $\Delta _{\rm SFR}$ to quantify ‘burstiness’ as a function of time, which is defined here as the logarithmic difference between the short-term averaged SFR (10 Myr) and the long-term averaged SFR (500 Myr). In the second panel from the top, we show that this parameter nicely captures the behaviour described above for the MW-mass object, displaying large values for a lookback time $\ge 10$ Gyr, but evolving smoothly afterwards. During this later, more quieter SFR phase, the circularity of the stars in the galaxy steadily increases (third panel top to bottom), signalling the build-up of the thin disc component during the smooth SFR phase. Here, circularity is calculated as the ratio between the *z*-component of the angular momentum of each star in a system rotated so that the total angular momentum of the stars points along the *z*-direction, compared to that of a circular orbit with the same energy ($\epsilon =j/j_{\rm circ}(E)$, M. G. Abadi et al. 2003). The bottom panel shows the age distribution of the stars selected to belong to the disc ($\epsilon >0.5$, cyan) or to the spheroid ($\epsilon < 0.5$, red), confirming the early formation of the stars that are today in the non-rotating component.

We extend this analysis to the regime of low mass galaxies, where the left and middle panels of Fig. 3 correspond to the discy and spheroidal dwarfs illustrated in Fig. 1. Contrary to MW-like galaxies, both dwarfs seem to maintain a substantial level of burstiness throughout their entire evolution. In the case of the discy dwarf (left), the mean circularity of the stars only recently ($\sim 2$ Gyr ago) starts to increase towards $\epsilon \sim 1$, indicating a much younger (and less dominant) disc than in the case of MW-like galaxies. There is also a subtle hint suggesting that the spheroidal dwarf has a larger degree of burstiness in its SFR overall, as quantified by a larger dispersion in $\Delta _{\mathrm{ SFR}}$ values during its entire time evolution.

**Figure 4.**
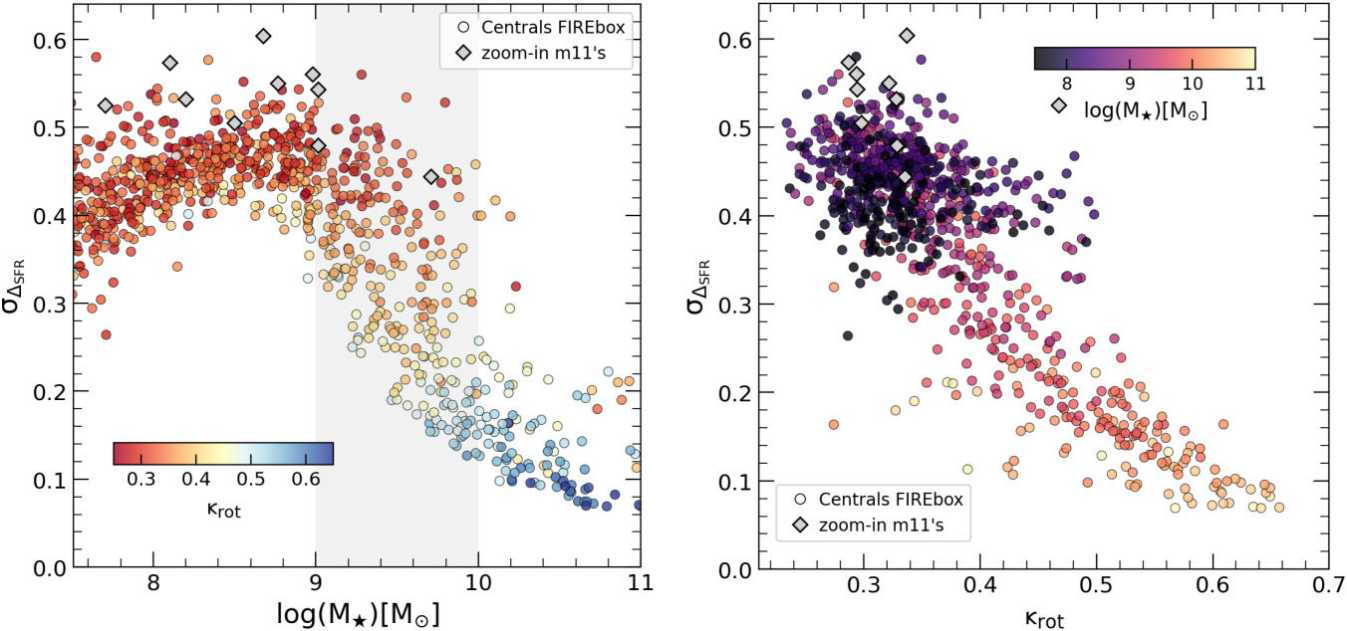
Left: burstiness as a function of stellar mass, where burstiness ($\sigma _{\Delta _{\rm SFR}}$) is quantified through the dispersion in the $\Delta _{\rm SFR}$ values averaged over the last 6 Gyr. Circles show all central galaxies in FIREbox colour codded according to their morphology ($\kappa _{\rm rot}$), blue are disc-dominated, red are spheroid-dominated. Zoom-in m11’s FIRE-2 runs are shown as before with grey diamonds, displaying a slightly higher burstiness than FIREbox objects (see the text). In FIREbox, burstiness shows a clear decline in the morphology ‘transition region’ (grey shaded), after which disc-dominated galaxies become common for $M_\star \ge 10^{10} ~ \rm {{\rm M}_{\odot }}$. At fixed stellar mass, galaxies with larger $\sigma _{\Delta _{\rm SFR}}$ are more dispersion-dominated (reddish colours) than those with lower burstiness, which tend to have more rotational support. Right: burstiness as a function of morphology in our sample, confirming a strong correlation between the two variables. Symbols are colour coded according to their stellar mass.

Individual inspection of these example galaxies suggest that the root mean square (r.m.s.) dispersion of the distribution of $\Delta _{\mathrm{ SFR}}$, or $\sigma _{\Delta _{\mathrm{ SFR}}}$, can be a helpful indicator of burstiness if integrated over a given time. Inspired by the results on the scale of the MW-like galaxies, we chose to analyse the late-time burstiness as the r.m.s. dispersion of $\Delta _{\mathrm{ SFR}}$ in the last 6 Gyr (range highlighted in green on the second row of Fig. 3). We note that the results presented below do not change qualitative if we instead vary this 6 Gyr cut-off by $\pm 2$ Gyr. We show on the left panel in Fig. 4 burstiness (quantified through $\sigma _{\Delta _{\mathrm{ SFR}}}$) as a function of stellar mass, or the $\sigma _{\Delta _{\mathrm{ SFR}}}-M_{\star }$ relation. Simulated galaxies are colour coded according to their morphology, with disc-dominated objects in blue and spheroid-dominated ones in red (see colour bar).

There is a very clear link between burstiness and morphology in our sample. Two factors are important to appreciate in the left panel of Fig. 4. First, the trend of burstiness with stellar mass: $\sigma _{\Delta _{\mathrm{ SFR}}}$ peaks at values 0.4–0.5 for stellar masses in the massive dwarf end, $M_\star \sim 10^8 \rm -10^9~\rm {{\rm M}_{\odot }}$, and systematically plunge downwards in the morphology ‘transition’ region (grey shaded region) reaching $\sigma _{\Delta _{\mathrm{ SFR}}} \sim 0.1$ at stellar masses comparable to the MW. Second, the scatter in burstiness at fixed stellar mass: galaxies with higher $\sigma _{\Delta _{\mathrm{ SFR}}}$ show more spheroidal-like components than low $\sigma _{\Delta _{\mathrm{ SFR}}}$ counterparts with similar stellar content. This effect is maximally visible in the morphology transition region (grey shaded), but is also clear for more massive, MW-like objects. Below $M_\star \sim 10^9 ~ \rm {{\rm M}_{\odot }}$ the trend disappears since the spread in morphologies is insufficient to display the effect. The strong relation between burstiness and morphology in our sample is further illustrated on the right panel of Fig. 4, where we show that $\sigma _{\Delta _{\mathrm{ SFR}}}$ is smoothly declining as morphologies become more disc-dominated. The overall trend of $\sigma _{\Delta _{\mathrm{ SFR}}}$ with $M_\star$ and its scatter at fixed stellar mass suggests that the FIRE-2 model predicts too bursty star formation histories to form and maintain rotationally supported stellar discs at low stellar masses. These results also nicely confirm the findings presented in S. Yu et al. (2023) based on FIRE-2 zoom-in runs of MW-like galaxies, and extend their validity over a wider range of masses, $10^9 < M_\star /\rm {{\rm M}_{\odot }} < 10^{11}$.

Grey diamonds in Fig. 4 suggest that the higher resolution zoom-in m11 runs roughly agree with results from FIREbox, although zoom-ins are located systematically at higher $\sigma _{\Delta _{SFR}}$. We have explicitly checked that re-computing $\sigma _{\Delta _{SFR}}$ from m11 runs using only an adjusted fraction of the particles to match the sampling in star formation history from FIREbox objects does not resolve the bias. Our definition of $\sigma _{\Delta _{\mathrm{ SFR}}}$ is relatively robust to the number of particles used to measure it, as far as more than ∼1000 stellar particles are used. Instead, we attribute this slightly higher $\sigma _{\Delta _{\mathrm{ SFR}}}$ in zoom-in runs to the ability of high resolution simulations to better represent stochasticity associated to star formation than lower resolution runs.

**Figure 5.**
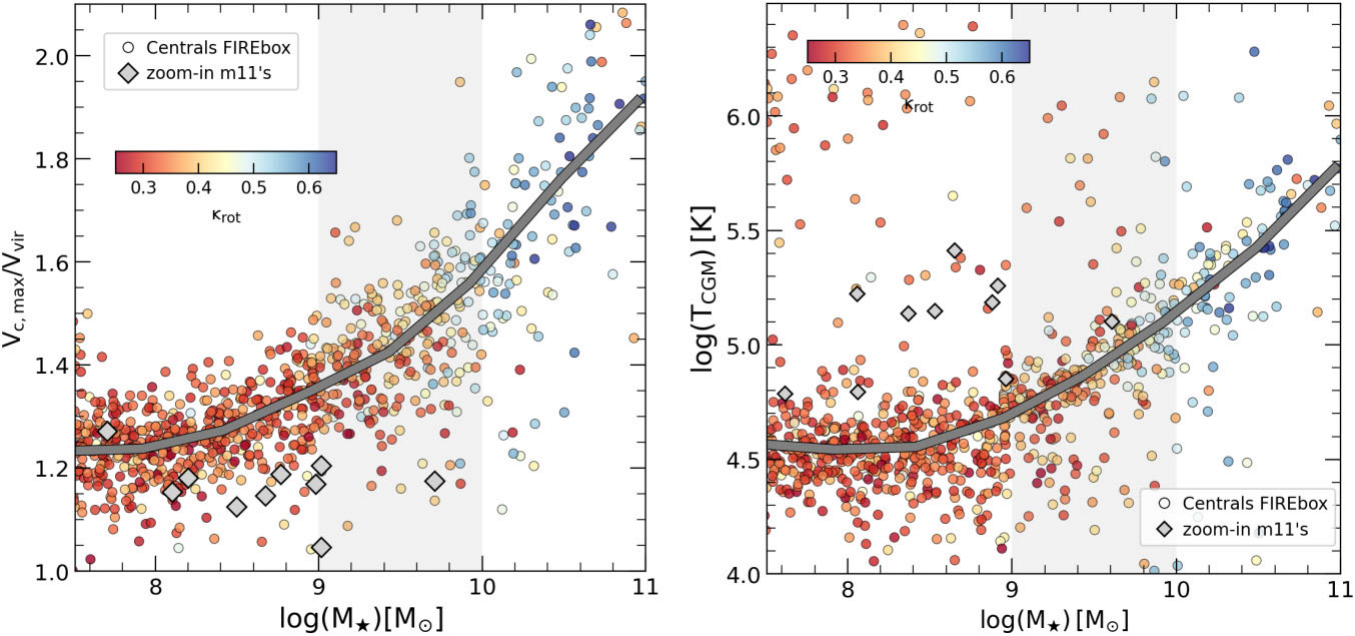
*Left*: depth of the potential well, here quantified as the ratio between maximum rotation velocity ($V_{\mathrm{ max}}$) and virial velocity of the halo ($V_{\mathrm{ vir}}$) calculated using all type of particles (stars + gas + DM), as a function of stellar mass for all central galaxies in FIREbox. Colour coding is the same as in Fig. 4 (see the text for discussion of *m11*’s runs). *Right*: median temperature of the CGM for each halo, calculated as the median temperature of all gas particles in the halo excluding the central regions ($0.3 < r/r_{\rm vir} < 1$). The gravitational potential depth and the temperature of the CGM steadily increases with stellar mass for $M_{\star } > 10^8 ~ \rm {M_\odot }$, facilitating the formation of discs for $M_\star \ge 10^{10} ~ \rm {{\rm M}_{\odot }}$.

Several factors coincide in time with the onset of a less bursty star formation history in objects with MW-like mass. Among them, a deepening of the gravitational potential of the halo (P. F. Hopkins et al. 2023) along with the establishment of a ‘virialized’ or hot CGM surrounding the central galaxy (J. Stern et al. 2021; Z. Hafen et al. 2022; A. B. Gurvich et al. 2023; S. Yu et al. 2023) have been reported to aid the settling of discs. We explore this in Fig. 5. In the left panel we use the ratio between the maximum circular velocity of the halo, $V_{\rm c,max}$, to the virial velocity, ($V_{\rm vir} = \sqrt{G M_{\rm vir}/r_{\rm vir}}$) to quantify the depth of the gravitational potential. On the right panel we show the median temperature of the CGM gas, defined here as all gas associated to the halo in the radial range $0.3 < r/r_{\rm vir} < 1$. Symbols are colour coded according to morphology ($\kappa _{\rm rot}$) and a thick grey line shows the median of the sample at a given $M_\star$.

We find that both, the velocity ratio and the temperature of the CGM monotonically increases with stellar mass, supporting a scenario where discs preferentially arise in haloes that are dense and can support a hot CGM. Note that the relation found between a hot CGM and the formation of discs differs from the classical picture where hot CGM is associated with red elliptical galaxies (e.g. A. Dekel & Y. Birnboim 2006). Instead a hot CGM promotes disc formation by supplying, through cooling, coherently aligned gas to feed the central gas disc (L. V. Sales et al. 2012; H. Übler et al. 2014). Low-mass haloes hosting dwarf galaxies have virial temperatures $T < 10^5\,\, \rm K$, making a stable hot CGM rather rare, helping explain the scarcity of discs in that regime (G. Zeng et al. 2024).

Of the factors explored in our FIREbox sample: burstiness (Fig. 4), potential depth, and CGM temperature (Fig. 5), burstiness shows a better and more direct correlation with individual morphologies for $M_\star > 10^9 ~ {\rm M}_{\odot }$, while potential depth and CGM temperature supports more of an average trend with stellar mass at least $> 10^8 ~ \rm {{\rm M}_{\odot }}$. As in Fig. 4, *m11* zoom-in runs tend to agree with results of FIREbox objects, although with a small bias towards lower gravitational potential in the inner regions (lower $V_{\rm max}/V_{\rm vir}$). This is consistent with a scenario where higher resolution zoom-in runs are able to resolve more of the bursty cycle of star formation leading to the formation of central dark matter cores that are slightly more pronounced than those formed in the lower resolution FIREbox dwarfs of similar mass.

For completeness, we have explored other ways to quantify the depth of the gravitational potential, obtaining similar results as shown above. For example, one can replace the maximum circular velocity by the circular velocity at a more internal radius, perhaps more connected to the physical scales of the gas and stars in galaxies. For this, we used the circular velocity measured at the fiducial radius $V_{\rm fid}=V_{c}(r_{\rm fid})$, where $r_{\rm fid}=2(V_{\rm max} /70 ~ \rm {km \, s ^{-1}})$ kpc as introduced in I. M. E. Santos-Santos et al. (2020), with typical values for $r_{\rm fid} \sim 1.3 - 9.5$ kpc for $M_\star \sim 10^8 \rm -10^{11} ~ {\rm M}_{\odot }$. The trend using $V_{\rm fid}/V_{\rm max}$ to quantify the potential depth is comparable to using $V_{\rm max}/V_{\rm vir}$ in Fig. 5.

### 3.3 Gas dynamics

**Figure 6.**
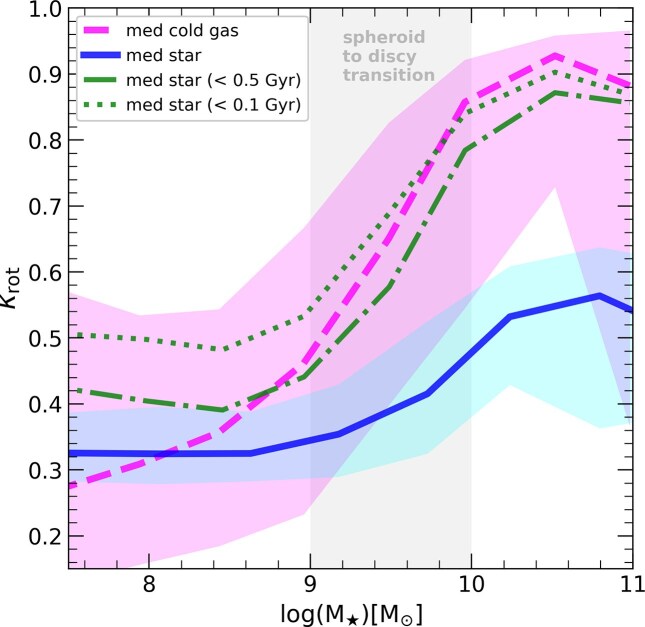
Rotational support of several galaxy components ($\kappa _{\rm rot}$) as a function of stellar mass. Pink dashed line shows the median of the $\kappa _{\rm rot}$ for the gas in all galaxies from FIREbox, along with the shaded region highlighting 10th–90th percentiles. Gas shows in general more rotational support than the stars (blue curve), but follows a similar trend: $\kappa _{\rm rot}$ values for the gas increase steeply in the ‘transition region’ (grey shaded) reaching almost perfect rotational support for MW-like objects. We show that young stars are born with similar orbits than the cold gas, shown here by the median $\kappa _{\rm rot}$ of stars younger than 500 Myr (dot–dashed) and 100 Myr (dotted green). Low mass galaxies, $M_\star < 10^9~ \rm {{\rm M}_{\odot }}$, show little rotational support in the stars because even for the gas component dispersion dominates the kinematics, explaining the lack of discs for the simulated dwarfs.

The absence of coherent rotational structure in galaxies can result from two different scenarios: (A) either stars initially form in a rotationally supported disc that is later disrupted (e.g. by mergers or misaligned gas accretion), or (B) stars never form in a rotationally supported configuration to begin with. We study this in Fig. 6, where we show $\kappa _{\rm rot}$ of the cold gas (pink) as a function of stellar mass. Here, the morphological parameter $\kappa _{\rm rot}$ is calculated as before (equation 1), but using cold gas elements within the galaxy radius instead of the stars, where cold refers to elements with temperature $T < 1.5 \times 10^4 ~ K$. The median of the population in FIREbox is shown by the long dashed curve, while shaded regions indicate the 10th–90th percentiles in the sample. The trend for the rotational support of the gas mimics closely that of the stellar morphology in simulated galaxies: low mass dwarfs have little rotational support on their gaseous component, which starts to steadily increase in the range $M_\star =10^9 \rm -10^{10} ~ {\rm M}_{\odot }$, reaching $\kappa _{\rm rot} \sim 0.9$ for MW-like galaxies. Note that while the trend is similar for gas and stars, stars (blue) shows substantially lower rotation than the gas component.

Results in Fig. 6 confirm the origin of the lack of discs in low mass simulated galaxies: the gas is unable to settle into rotationally supported components from which stars can be born (T. Kaufmann, C. Wheeler & J. S. Bullock 2007). This is further demonstrated by the green solid and dotted lines, showing the median $\kappa _{\rm rot}$ of stars that are younger than 0.5 and 0.1 Gyr, respectively. Unsurprisingly, the morphology of young stellar populations follows that of the cold gas that fuels their formation. In low mass objects with $M_\star < 10^9 ~ \rm {{\rm M}_{\odot }}$ the absence of gas discs leads to the formation of stellar components that lack rotational support. This rules out scenario (A) above, where the lack of discs is explaining by stars born in misaligned discs. Rather, the lack of discs for simulated galaxies with $M_\star < 10^9 ~ \rm {{\rm M}_{\odot }}$ in FIREbox can be attributed to the turbulent nature of the gas in those systems and directly linked to the coupling of stellar feedback to the ISM in dwarfs (T. Kaufmann et al. 2007).

**Figure 7.**
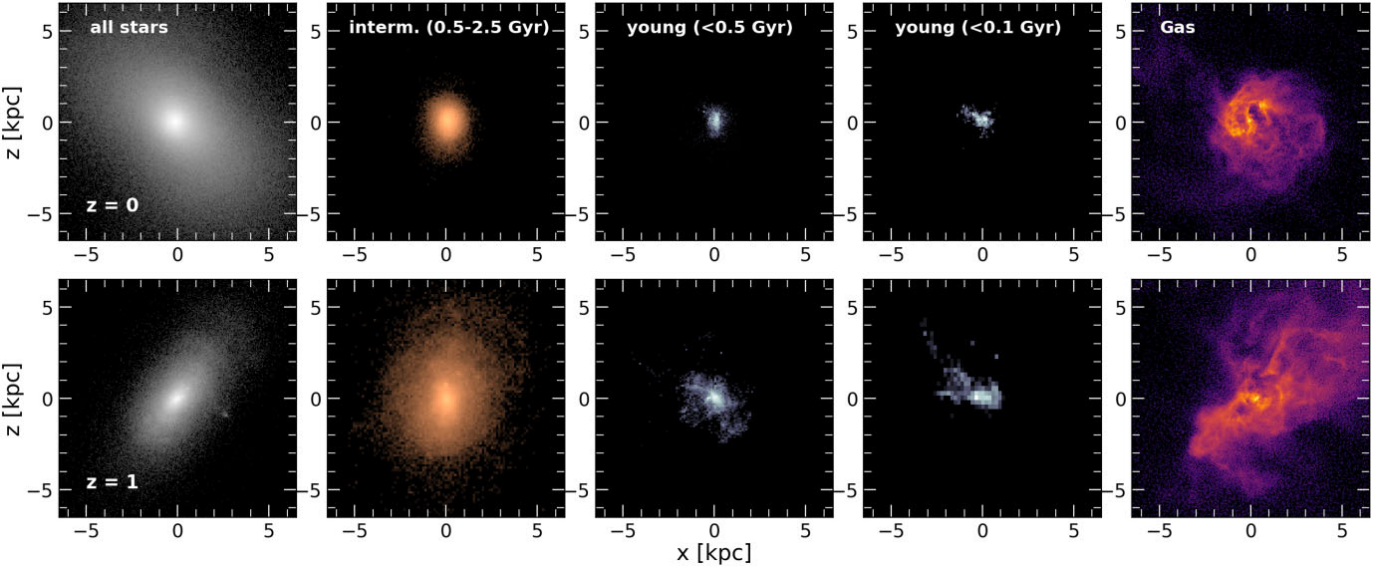
Projections of the stellar (first four columns) and gas (fifth column) components at redshift $z=0$ (top row) and $z=1$ (bottom row) for the *m11d UVB 880* zoom-in run. In the first column, we include all stellar particles, and we divide them by age into intermediate (0.5–2.5 Gyr), and young ($< 0.5$ or $< 0.1$ Gyr) in the subsequent columns. Gas cell distribution is shown in the last column. In each column, we show the edge-on projection, where the system has been rotated with the angular momentum of the displayed stellar particles (or gas cells) pointing along the *z* direction. In all cases, the structure is irregular and turbulent, with little ordered rotational support and no disc.

**Figure 8.**
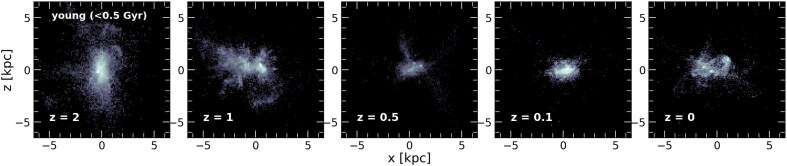
Edge-on projections of the younger stellar population (age <0.5 Gyr) in the *m11q MD 880* zoom-in run at different redshifts (from left to right) $z=[2,1,0.5,0.1,0]$. As the other example shown in Fig. 7, this simulation never develops a disc structure throughout time, with their young stars forming with irregular morphologies since $z=2$.

Fig. 7 illustrates this point further using the higher resolution *m11d UVB 880* zoom-in run (a spheroidal low-mass galaxy with $\kappa_{\rm {rot},z=0} \sim 0.29$). The top row corresponds to the present-day $XZ$ projections of (from left to right) all stars, intermediate, young and very-young stars and gas. Each panel has been individually rotated so that the *z*-component of the angular momentum of each sub-set of particles is pointing along the *z*-axis (i.e. these correspond to ‘edge-on’ views). As is clear from these projections, no axisymmetric disc-like component exists at $z=0$. This statement is also true of an earlier time, $z=1$, shown in the bottom row of the same figure. Moreover, in Fig. 8 we select another high-resolution zoom-in run, the *m11q MD 880* ($\kappa_{\rm {rot}, z=0} \sim 0.34$) halo with quietest merger history, and show the edge-on view of the young stars (age $< 0.5$ Gyr) at redshifts $z=2, 1, 0.5, 0.1$ and 0 (left to right). At all times, young stars are born in turbulent structures with no clear sense of coherent rotation.

Our results agree well with previous conclusions in K. El-Badry et al. (2018a), highlighting the turbulent nature of the gas in FIRE zoom-in runs of low mass galaxies, and extend their conclusions to a much larger volume-complete sample from FIREbox. We also demonstrate that this is not a feature of the present-day universe, but rather turbulent structures prevail during most of the cosmological evolution of these dwarfs. On the other hand, for more massive galaxies, once the gravitational potential is able to provide support to contain the bursty cycles, gas morphology becomes dominated by rotation and stellar discs comparable to those of the MW are commonly formed. In other words, the ‘morphological transition’ identified in Fig. 1, is a reflection of the mass range where the treatment of the physical processes included in the simulation allows for a transition from turbulent-dominated ISM ($M_\star < 10^9 ~ \rm {{\rm M}_{\odot }}$) to ordered rotation ($M_\star \ge 10^{10} ~ \rm {{\rm M}_{\odot }}$).

**Figure 9.**
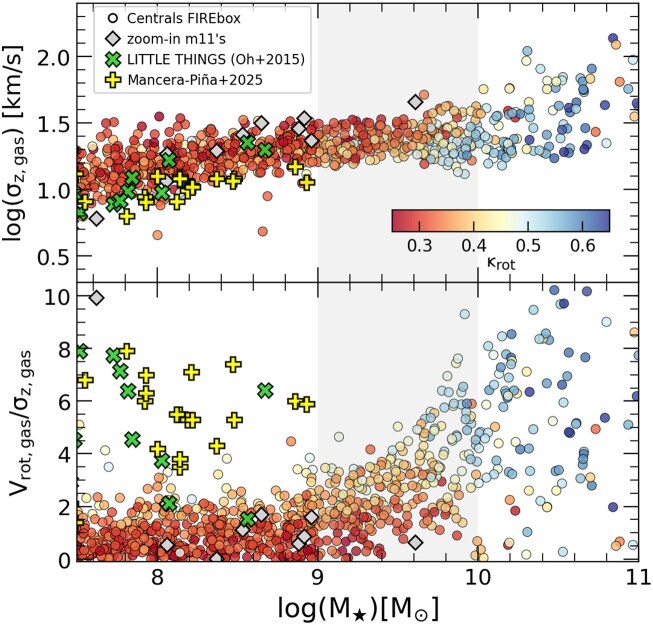
Top: *z*-component of the velocity dispersion in the gas for FIREbox simulated central galaxies colour coded by the morphology parameter, $\kappa _{\rm rot}$. The velocity dispersion shows a weak dependence in stellar mass, with $\sigma _{z,\rm gas}$ ∼ 10–20 km s^–1^ below $M_\star \sim 10^{10}~ \rm {{\rm M}_{\odot }}$. Bottom: ratio of gas rotational velocity to velocity dispersion, $\rm {V_{rot}/\sigma _z}$, as a function of stellar mass. In agreement with the morphological indicator $\kappa _{\rm rot}$, rotational support as measured by $V/\sigma$ in simulated galaxies start growing above 1 in the ‘transition region’ with $M_\star =[10^9 \rm -10^{10}]~$$\rm M_\odot$, but remains at very low values for the dwarfs due to the minimum floor in $\sigma _z$. For comparison, we show observational measurements from the rotation curve modelling in 10 LITTLE THINGS galaxies (S.-H. Oh et al. 2015) and the sample of dwarfs galaxies from P. E. Mancera Piña et al. (2025), indicated with green and yellow crosses, respectively, which suggest that simulated dwarfs have gas components with larger degree of turbulence than in this sample.

Challenging these results, observations show the formation of rotationally supported discs at lower masses than the transition region in our simulations. Velocity profiles of H i gas remain double-peaked in observations for velocities where models like FIRE predict a single Gaussian-like distribution (K. El-Badry et al. 2018b). Similarly, the stellar shape distributions suggest an excess of spheroid-like dwarfs in FIREbox (C. Klein et al. 2025). We investigate this in our sample in Fig. 9, where we show the gas velocity dispersion as a function of stellar mass (top panel) along with the rotational velocity to dispersion ratio (bottom panel). Note that careful comparisons to observational data in the Local Group indicates that the FIRE model reproduces nicely the observed gas dynamics in galaxies above the morphology transition region, or $M_\star \ge 10^{10}$$\ \rm M_\odot$ (F. McCluskey et al. 2024, 2025).

The top panel of Fig. 9 shows that below such scale the velocity dispersion varies only weakly with stellar mass, with characteristic values $\sigma _{z,\rm gas}$ ∼ 10–20 km s^–1^ for simulated $M_\star < 10^{10} ~ \rm {{\rm M}_{\odot }}$ objects. For comparison, we include the average velocity dispersion calculated by the modelling of the rotation curve in a sample of observed dwarf galaxies from the Local Irregulars That Trace Luminosity Extremes, The H I Nearby Galaxy Survey (LITTLE THINGS) (S.-H. Oh et al. 2015) and P. E. Mancera Piña et al. (2025, hereafter MP25), indicated with green and yellow crosses, respectively.^2^ The velocity dispersion in the simulation is in reasonable agreement, although there is evidence of a systematically smaller dispersion in LITTLE THINGS for $M_\star < 10^8$$\ \rm M_\odot$. Note that this is a conservative comparison, as the velocity dispersion of the gas in the simulations is calculated only on the *z*-component after each galaxy has been rotated so that the total angular momentum is aligned with the *z*-axis, and not the projected line-of-sight component resulting from the total 3D dispersion.

The bottom panel of Fig. 9 shows the ratio between the rotation velocity $V_{\rm rot,gas}$ to the predicted velocity dispersion $\sigma _{z, \rm gas}$ in the simulations. Here, we calculate $V_{\rm rot,gas}$ in the rotated framework of each galaxy with their stellar angular momentum pointing along the *z*-direction and assign the rotation velocity to the azimuthal component of the velocity


(3) \begin{eqnarray*}
V_{\rm rot} = V_\phi = \frac{x v_y - y v_x}{\sqrt{x^2 + y^2}} \ ,
\end{eqnarray*}

Once we have both radial profiles ($V_{\rm rot}, \sigma _z$) we measure the ratio between the maximun of the $V_{\rm rot,gas}$ and the median of $\sigma _z$, both within 0.15 $\ r_{\rm vir}$. The bottom panel in Fig. 9 illustrates that only galaxies with $M_\star > 10^9~ \rm {{\rm M}_{\odot }}$ are massive enough to have $V_{\rm rot,gas} \ge 3\sigma _{z,\rm gas}$, giving raise gradually to the formation of rotationally supported discs in the morphology transition region $M_\star =10^9 \rm -10^{10}~ \rm {{\rm M}_{\odot }}$ and beyond. This introduces a minimum halo mass roughly $M_{\rm vir} \sim 10^{11}~ \rm {{\rm M}_{\odot }}$ where discs can form. Instead, most of the LITTLE THINGS and MP25 galaxies (green and yellow crosses) show $V_{\rm rot,gas}/\sigma _{\rm gas} > 3$ despite their low-stellar mass (and inferred low dark matter halo). While LITTLE THINGS and MP25 are not representative of the majority of dwarfs (see discussion below), Fig. 9 demonstrates the existence of real low mass galaxies with substantially larger rotation support than the ones formed within FIREbox.

**Figure 10.**
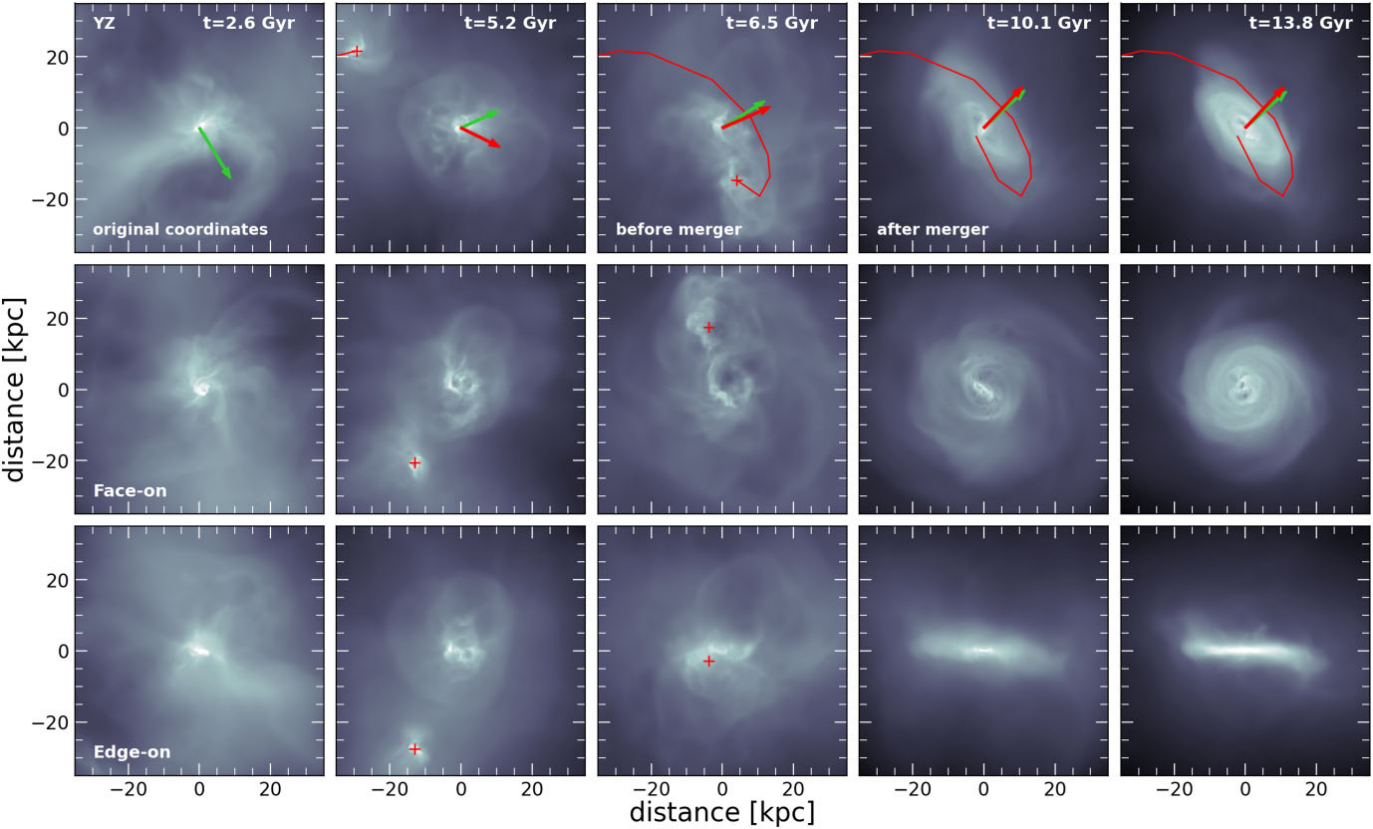
Time evolution for the formation of a disc component in the *m11b* zoom-in run. Top row: each panel shows the gas distribution at different times (columns) in the YZ projection using the original coordinates of the simulation. The light-green arrow indicates the angular momentum vector of the gas (calculated with the gas cells within $r_{\mathrm{ gal}}$), while the red arrow shows the angular momentum vector of the orbit of a satellite responsible for bringing in the gas that forms the rotationally supported disc. We also indicate its orbit with a red solid line for some times before and after the merger. Middle and bottom rows present similar information but rotated to show the face-on and edge-on projections of the system such that the angular momentum of the central galaxy at each time points along the *z*-axis. The position of the satellite is highlighted by the red cross before the merger. The interaction with this gas rich companion mostly explains the formation of a large gas disc in this dwarf, which then fuels the formation of a stellar disc with significant rotation, explaining why *m11b* is a high $\kappa _{\rm rot}$ outlier in the morphology–stellar mass relation (Fig. 1).

Besides the shallow $\sigma$–$M_\star$ relation below $M_\star \sim 10^{10} ~ \rm {{\rm M}_{\odot }}$ highlighted in the top panel of Fig. 9, there is an additional contribution to the low rotation velocity in our simulated galaxies: the halo mass–stellar mass relation. Fig. 10 in R. Feldmann et al. (2023) indicates that simulated dwarfs in FIREbox occupy smaller dark matter haloes than expected from available abundance matching relations. With lower $M_{200}$ at a given stellar mass, the rotational velocity (or circular velocity) is expected to be lower than galaxies that inhabit a larger mass dark matter halo, contributing partially to the low $V/\sigma$ ratios in the low mass simulated galaxies.

It is important to keep in mind that comparisons between simulations and observations of gas and star kinematics in the regime of dwarfs is challenging. Samples like LITTLE THINGS and MP25 are heavily biased towards finding rotation (since they are selected for kinematical studies and dynamical modeling) and are far from representative of the overall dwarf population. Our results suggest that simulated dwarf galaxies show less rotation than *some* observed dwarfs, for example, from the LITTLE THINGS survey or MP25 sample. But this should not be interpreted as a general inadequacy on the structure predicted for *all* simulated dwarfs. On the contrary, FIRE-2 and FIREbox runs have been shown to reproduce several of the scaling relations of observed in low-mass systems. For example, C. Klein et al. (2024) shows that synthetic mock observation of simulated galaxies in FIREbox are in good agreement with the observed mass–size relation. Similarly, the gas kinematics in FIRE-2 dwarf zoom-in runs is also shown to overlap with many of the observed dwarfs when comparing unresolved H i profiles (K. El-Badry et al. 2018b), including a good agreement with the observed baryonic Tully–Fisher relation. The reader is referred to section 8.2 in K. El-Badry et al. (2018a) and sections 4.1 and 4.2 in K. El-Badry et al. (2018b) for a detailed discussion of biases in observations and simulations that should be kept in mind when comparing.

What our work highlights here is that the same galaxy formation model, when applied in different regimes, predicts a very different typical structure for galaxies: very commonly disc-dominated in the scale of MW-mass galaxies, but increasingly dominated by dispersion as stellar mass falls below $M_\star \sim 10^8~ \rm {{\rm M}_{\odot }}$. This is a robust prediction that agrees well with observations: dwarf galaxies are found to be rounder and the discs turn thicker at the low mass end (A. Helmi et al. 2012; S. Roychowdhury et al. 2013; R. C. Simons et al. 2015; M. C. Johnson et al. 2017). Fig. 9 only highlights that the Universe is able to create *at least some* dwarf galaxies that are rotationally supported in the low mass regime where FIREbox tends to form systems with significant dispersion support. How common such discy dwarfs are in the Universe is still unclear and therefore it might require of simulations of larger volumes to generate a comparable system within the FIRE model. A more clear assessment of the severity of disc scarcity in the regime of dwarfs and the exact scale (stellar mass) where this becomes a problem can only be achieved once kinematical studies of volume-complete samples of low mass galaxies become available.

A similar transition between velocity dispersion to rotational support was reported by B. Clauwens et al. (2018) using the Evolution and Assembly of GaLaxies and their Environments (EAGLE) simulations. Interestingly, the stellar mass range where the morphology of galaxies change coincides well with the one found here for FIREbox. More recently, B. M. Celiz et al. (2025) also reported a mass–morphology relation (see Fig. 1) in the TNG50 simulation, but with a ‘transition region’ occurring at lower stellar masses, $10^8 < M_\star /\rm {M_\odot } < 10^9$. In the low-mass end of that range, G. Zeng et al. (2024) finds that TNG50 dwarfs form their stars in discs, but later episodes of star formation occur in gas discs that are misaligned with previous events, leading to mostly predictions of dispersion-dominated objects for their low mass dwarfs. While the mass–morphology trend in TNG50 is similar to the one found in our sample, the exact nature of the lack of rotationally supported discs in dwarfs seems different than in FIREbox. B. M. Celiz et al. (2025) find that low mass galaxies form the majority of their stars in a central unresolved clump, explaining their lack of stars with ordered rotational support. The authors mention that the central clumps are connected to shortcomings of the wind model and the assumption of a hydrodynamically decoupled wind. In contrast, FIREbox includes a completely different treatment of winds where deposition is local and is able to generate self-consistent outflows and a multiphase interstellar medium. We have also shown that in FIRE, dispersion-dominated dwarfs tend to form their stars not in misaligned discs that cancel their angular momentum, but directly from dispersionally supported gas.

In the case of FIREbox, other factors might come into play to explain the increase of dispersion support for low mass galaxies. For example, comparison with observations seem to suggest that the time-scale between bursts of star formation in the FIRE model could be too short for intermediate mass dwarfs with $M_\star \sim 10^8~ \rm {{\rm M}_{\odot }}$ (N. Emami et al. 2019). This would result in more active bursty cycles in low mass galaxies, resulting in a higher amount of energy coupling from stellar feedback into the interstellar medium and, therefore, larger expected degree of gas turbulence, in agreement with our detected trend in FIREbox. Other codes have also reported effects on morphology of dwarf galaxies related to the details of the sub-grid physics model. For instance, runs from the Marvel-ous suite (F. Munshi et al. 2021) also suggest that two different models for supernova energy deposition (blastwave versus superbubble feedback) would lead to differences in the burstiness expected for low mass galaxies, albeit in their simulations, the feedback-related changes in the bursty cycles appear only for lower mass objects than here, closer to the regime of ultrafaint dwarfs. E. Zhang et al. (2024) shows using the Stars and MUltiphase Gas in GaLaxiEs (smuggle) model that the directionality of the winds assumed can have an impact on the level of burstiness in a $M_\star \sim 10^8~ \rm {{\rm M}_{\odot }}$ dwarf and also its gas and stellar morphology (in addition to its inner dark matter profile). We conclude that, given the strong relation between star formation cycles, the generated stellar feedback, and which fraction of it effectively couples to the surrounding gas, the theoretical predictions for the morphology of dwarf galaxies in galaxy formation simulations are still uncertain, and heavily impacted by the choices and limitations in the numerical implementation of feedback and star formation.

## 4 A DISC IN A LOW-MASS DWARF: THE CASE OF *m11b*

The left panel in Fig. 3 shows that one of the FIRE-2 high resolution zoom-in runs used here, *m11b MD + CR 2100* (hereafter *m11b*), displays a significant amount of rotational support ($\kappa _{\rm rot} \sim 0.47$) that is well above the median $\kappa _{\rm rot} \sim 0.3$ seen in counterparts with similar stellar mass $M_{\star } \sim 4.2 \times 10^{7} ~ \rm {M_\odot }$ and even the $\kappa _{\rm rot}$ for the other m11 zoom-in runs that are more massive. This raises the question as to the nature of the rotational support found in the *m11b* run. Note that a similar version o this *m11b* run, with standard FIRE-2 physics, has been previously studied (see e.g. T. K. Chan et al. 2018; K. El-Badry et al. 2018a,b) and is also the main object modified and studied in P. F. Hopkins et al. (2023). The analysis of *m11b* presented here uses a version with additional physics, including metal diffusion and cosmic rays. However, we have checked that the stellar mass and morphology in this particular run are very close to what was obtained with the more traditional flavour of the FIRE-2 model. For example, using the metal-diffusion run, we find $M_{\star }= 5.0 \times 10^{7} ~ \rm {{\rm M}_{\odot }}$ and $\kappa _{\rm rot}=0.46$, in good agreement with the values in our *m11b MD + CR 2100* run. This confirms that the additional physics included may not fundamentally alter the assembly and resulting morphology in this object, which is the target of study below.

We tracked the evolution of the *m11b* host halo and identified a satellite interaction about $\sim$8.5 Gyr ago ($z \sim 1.2$). The satellite has a stellar mass $M_{\star ,\rm sat} \sim 6 \times 10^{6} ~ \rm {{\rm M}_{\odot }}$ and is gas-rich ($M_{\rm gas,sat} \sim 3 \times 10^{7} ~ \rm {{\rm M}_{\odot }}$). At this redshift, the stellar mass of the *m11* main host galaxy is $M_{\star ,\rm host} \sim 3.3 \times 10^{7} ~ \rm {{\rm M}_{\odot }}$, meaning that the satellite interaction corresponds to a stellar mass ratio $M_{\star , \rm sat}/M_{\star , \rm host} \sim 0.2$. This interaction seems to have a significant impact on the posterior evolution of the central dwarf. We show this in Fig. 10, which shows the gas distribution within a box of 60 kpc centred at the main galaxy in the *m11b* run. Different columns correspond to different times throughout its evolution, including before and after the merger at $t_{\rm merger} = 7.5$ Gyr. The top row corresponds to the distribution of the gas cells, in the $YZ$ projection, by using the original coordinates of the simulation; while the middle and bottom rows show the face-on and edge-on projections, respectively, with the system rotated such that the total angular momentum points along the *z*-axis.

The top row illustrates that the interaction occurs such that the orbital momentum of the companion is roughly aligned with the angular momentum of the gas in the central dwarf. To visualize this, we use green arrows in the top row panels to indicate the direction of the total angular momentum of the gas of the central galaxy while the red arrow indicate the direction of the angular momentum of the orbit of this satellite companion. We also use a solid red line to show the projected orbital path followed by the satellite before it merges completely at $t \sim 7.5$ Gyr. For the last two columns, we freeze the red line and arrow to those measured at the last snapshot where the satellite is identified before merging. The special alignment of this interaction means that the satellite delivers a substantial amount of gas with large amounts of angular momentum to this object. The middle and bottom rows in Fig. 10 highlight that as a result of this interaction, a very well defined gas disc is formed, which is particularly thin and extended up to a radii ∼20 kpc as a result of the increased angular momentum content originated from the coincidental alignment of the satellite orbit (red crosses highlight the position of the companion in snapshots where it can still be identified by the halo finder). After the disc is formed, star formation occurs in the disc, increasing the $\kappa _{\rm rot}$ of the stars in this galaxy and explaining their excess rotational support compared to other objects in FIREbox or FIRE-2 zoom-in runs. While such interactions are rare in $\Lambda$CDM, this case nicely reminds us that extended rotationally supported discs may occur even in dwarfs as low mass as $M_\star \sim 10^7~ \rm {{\rm M}_{\odot }}$ and the frequency of them in observations may provide helpful constraints for the sub-grid models in galaxy formation simulations. Note that our results align well with previous studies highlighting disc formation in low mass galaxies as a result of specially aligned mergers (e.g. B. Robertson et al. 2006; G. Zeng, L. Wang & L. Gao 2021; X. Wu et al. 2025).

## 5 SUMMARY

In this study, we explore the morphology of galaxies in the stellar mass range $M_\star =[10^{7.5} \rm -10^{11}]~ \rm {{\rm M}_{\odot }}$ using the FIREbox simulation (R. Feldmann et al. 2023), with particular emphasis on the regime of dwarf galaxies. We also use several higher resolution zoom-in runs from the *m11*’s suite (halo mass $M_{\rm vir} \sim 10^{11} ~ \rm {{\rm M}_{\odot }}$) also run with the FIRE-2 model to study individual objects with a higher time-cadence and larger numerical resolution. We characterize morphology using the dynamical criteria $\kappa _{\rm rot}$ which measures the fraction of the kinetic energy in ordered rotation (L. V. Sales et al. 2012).

Overall, we find that FIREbox is able to reproduce a variety of morphologies in the galaxy population, ranging from dispersion-dominated spheroidal systems to rotational-supported thin discs. We identify a clear trend between morphology and stellar mass that supports the idea that low mass galaxies are dispersion-dominated while galaxies comparable to the MW often host dominant stellar discs. In particular, we find that low mass dwarfs with $M_\star < 10^9~ \rm {{\rm M}_{\odot }}$ are rarely disc-dominated, while more massive galaxies with $M_\star > 10^{10}~ \rm {{\rm M}_{\odot }}$ are mostly rotationally supported. Therefore, FIREbox predicts a ‘transition regime’ from $M_\star =10^9 \rm -10^{10}~ \rm {{\rm M}_{\odot }}$, where discs start to arise and become the dominant dynamical component for galaxies of mass comparable to the MW. Some evidence exists in support of a similar transitional mass in observations (R. C. Simons et al. 2015).

We investigate the physical factors that determine this morphology trend with stellar mass in our simulations. We find that, contrary to galaxies of $\sim$MW-mass, the spin of the halo can play a significant role in the rotational support of the stellar component in low mass galaxies ($M_\star < 10^{10}~ \rm {{\rm M}_{\odot }}$), in agreement with previous claims using different simulations and baryonic treatment (e.g. V. Rodriguez-Gomez et al. 2017). However, there is no indication that halo spin plays a role in establishing the morphology transitional mass or the overall trend of morphology with stellar mass.

Instead, we find that the burstiness of the star formation history (here characterized by $\sigma _{\Delta _{\rm SFR}}$, Fig. 4) shows a strong correlation with both, the morphology–mass relation as well as its scatter at fixed stellar mass. In other words, in FIREbox, galaxies of a given stellar mass show higher rotational support in their stars (high $\kappa _{\rm rot}$) if their star formation histories are less bursty than counterparts showing more violent star formation cycles. In particular, measured as the r.m.s dispersion of the star formation history averaged in 0.1 and 0.5 Gyr, the $\sigma _{\Delta _{\rm SFR}}$ parameter shows a strong decline in the stellar range $M_\star =[10^9 \rm -10^{10}]~ \rm {{\rm M}_{\odot }}$, coincident with the scales where morphology globally transitions from purely dispersion dominated to mostly disc-dominated. Additionally, we find that the depth of the potential well along with the presence of a hot CGM accompanies the trend in burstiness, showing a systematic increase with stellar mass that follows the occurrence of discs in the simulation.

Rotationally supported stellar discs become increasingly rare at low masses in the simulation due to the predicted dispersion support in the gas component, which is tied to the star formation burstiness. The gas velocity dispersion scales only weakly with stellar mass, with lower bound values $\sigma _z \sim 10$ km s^–1^ for $M_\star =10^8 ~ \rm {{\rm M}_{\odot }}$ and only ∼20 km s^–1^ for masses $M_\star > 10^{10.5}~ \rm {{\rm M}_{\odot }}$. At the typical rotational velocities in this mass range (average $V_{\rm rot}= 10$ km s^–1^ and $>160$ km s^–1^, respectively), this corresponds to a gas rotational support $V/\sigma \sim 0.5$–1 for low mass objects (and therefore, mostly dispersion-dominated systems), while galaxies beyond the ‘transitional mass’ $M_\star > 10^{10}~ \rm {{\rm M}_{\odot }}$ may display substantially more rotational support $V/\sigma \sim 1$–9. Stars are born from the gas and therefore inherit its kinematics, explaining the morphological trend with mass in our sample. Only when galaxies occupy sufficiently massive haloes (deep potential wells), gravity is able to contain the effects of the bursty star formation cycles, providing an avenue to stabilize gaseous discs and start growing a stellar component with ordered rotation. Our results agree well with previous studies of the collaboration characterizing galaxy morphology using FIRE-2 zoom-in runs (J. Stern et al. 2021; S. Yu et al. 2021, 2023; Z. Hafen et al. 2022; A. B. Gurvich et al. 2023; P. F. Hopkins et al. 2023 ), and extend the analysis to include a continue range of stellar masses, different environments and assembly histories as sampled by the cosmological volume box in FIREbox.

In our simulations, the morphological transition occurs for $M_\star =[10^9 \rm -10^{10}]~ \rm {{\rm M}_{\odot }}$, which means that MW-like galaxies are often predicted to host dynamically dominant thin discs, but they become rare for $M_\star < 10^9~ \rm {{\rm M}_{\odot }}$. A similar mass–morphology trend is inferred from observations (S. Roychowdhury et al. 2013; R. C. Simons et al. 2015), although the exact mass scale for the transition is not yet well constrained. The lack of volume-complete observational samples at the faint-end also prevents a proper sampling of the dispersion around the median of the mass–morphology relation and a better understanding of the rotation- and dispersion-dominated extremes. For example, a comparison with LITTLE THINGS and MP25 suggests that rotationally supported dwarfs with $V/\sigma >3$ do exist for real galaxies with $M_\star \sim 10^8~ \rm {{\rm M}_{\odot }}$, which are absent in our sample. However, selection effects in observational dynamical studies prevents a thorough evaluation of the degree of the disagreement: LITTLE THINGS objects were selected based on their large rotation. Previous studies have found that the FIRE-2 model may overpredict the gas dispersion support in the low mass end (K. El-Badry et al. 2018a,b). In our case, we find that in addition to a slightly higher velocity dispersion in FIREbox compared to low-mass LITTLE THINGS galaxies, the low rotation velocity (itself associated to their low mass dark matter haloes) also contributes to the low $V/\sigma \le 1$. Models where star formation proceeds less efficiently and dwarfs occupy larger dark matter haloes at fixed $M_\star$ – as perhaps predicted by current abundance matching models – may help form some more rotationally supported discs in the regime of dwarfs.

Recently, studies using the TNG50 simulations also reported a similar morphology-stellar mass trend, albeit their transition region occurs at lower masses, closer to $M_{\star} \sim 10^8 \ {\rm M}_{\odot}$ (G. Zeng et al. 2024; B. M. Celiz et al. 2025). TNG50 and FIRE are very different models, and the origin of the lack of discs in TNG50 seems unrelated to the one found here for FIREbox. Other simulations have also reported substantial changes in morphology or bustiness in the dwarfs regime triggered by variations in the treatment of feedback (e.g. B. Azartash-Namin et al. 2024; E. Zhang et al. 2024). While most of these models have successfully reproduced the morphology of galaxies at the scale of the MW, the extrapolation of the sub-grid models to the regime of dwarfs highlights larger uncertainties (or disagreement) in this regime. The morphology of low-mass galaxies may therefore provide a more refined test of our galaxy formation models, extending beyond the successes already achieved in understanding the formation of MW-mass discs.

## Data Availability

This paper is based on snapshots, halo catalogues, and merger trees from the FIRE zoom-ins (P. F. Hopkins et al. 2014, 2018b) and FIREbox (R. Feldmann et al. 2023) data. Some public data are available at https://fire.northwestern.edu/. The FIRE-2 zoom-in simulations are publicly available (A. Wetzel et al. 2023) at FlatHUB. The main properties of the galaxy samples, and other products included in this analysis, may be shared upon request to the corresponding author if no further conflict exists with ongoing projects.

## Footnotes

1
https://fire.northwestern.edu/

2 For the observations from LITTLE THINGS, we have calculated the average $\sigma$ (azimuthally averaged H i velocity dispersion) from column $b)$ of their reported asymmetric drift corrections for each individual rotation curve model, while for MP25 they got $V_{\rm {rot}}$ and $\sigma _{\rm {HI}}$ using barolo (E. M. Di Teodoro & F. Fraternali 2015). We include only dwarfs with $M_\star < 10^9 ~ \rm { {\rm M}_{\odot }}$
